# The Optical Signal-to-Crosstalk Ratio for the MBA(*N*, *e*, *g*) Switching Fabric †

**DOI:** 10.3390/s21041534

**Published:** 2021-02-23

**Authors:** Remigiusz Rajewski

**Affiliations:** Faculty of Computing and Telecommunications, Institute of Communication and Computer Networks, Poznan University of Technology, Polanka 3, 60-965 Poznan, Poland; remigiusz.rajewski@put.poznan.pl

**Keywords:** crosstalk, MBA, optical, OSXR, switching fabric, switching network

## Abstract

The *banyan*-type switching networks, well known in switching theory and called the $\log_{d}N$ switching fabrics, are composed of symmetrical switching elements of size d×d. In turn, the modified baseline architecture, called the MBA(N,e,g), is only partially built from symmetrical optical switching elements, and it is constructed mostly from asymmetrical optical switching elements. Recently, it was shown that the MBA(N,e,g) structure requires a lower number of passive as well as active optical elements than the *banyan*-type switching fabric of the same capacity and functionality, which makes it an attractive solution. However, the optical signal-to-crosstalk ratio for the MBA(N,e,g) was not investigated before. Therefore, in this paper, the optical signal-to-crosstalk ratio in the MBA(N,e,g) was determined. Such crosstalk influences the output signal’s quality. Thus, if such crosstalk is lower, the signal quality is better. The switching fabric proposed in the author’s previous work has lower optical signal losses than a typical Beneš and *banyan*-type switching networks of this same capacity and functionality, which gives better quality of transmitted optical signals at the switching node’s output. The investigated MBA(N,e,g) architecture also contains one stage fewer than *banyan*-type network of the same capacity, which is an essential feature from the optical switching point of view.

## 1. Introduction

In many elaborations from the last few years, it can be seen that the number of users of desktop applications grows about 50 million each year while the number of people using mobile apps grows more than 200 million each year [1]. A visible result of the increase in the number of application users is an increase in the volume of data sent through networks and processed by servers. It also fits in the prediction of the Cisco company—one of the network equipment tycoons. Cisco publishes annually special reports estimating the network traffic expressed by the visual networking index (VNI). Cisco predicts that the global network traffic will increase from 1.5 ZB in the year 2017 to 4.8 EB in the year 2022 [2]. This means that network traffic will be increased three times. Thus, more resources are needed to handle higher traffic, for example, more or faster links, more routers, and more switching capacity (larger switching nodes in size nodes) [3]. Because of the Compound Annual Growth Rate (CAGR, years 2015–2020), IP traffic will be equal to 22% [4], such an approach to sustaining the IP core network expansion might not be enough. The annual global IP traffic at the end of 2016 already exceeded the zettabyte threshold [4]; therefore, new ways of increasing network throughput are required. Nowadays, there is no problem to add more links to the used networks or to using quicker links. However, extending the switching node became sometimes a massive problem due to the complexity of routing, resources management, and even the number of physical elements needed to construct such a node, especially the optical switching node. The node is becoming a bottleneck for switching. Therefore, new conceptual and technological solutions are introduced.

**Figure 1:**
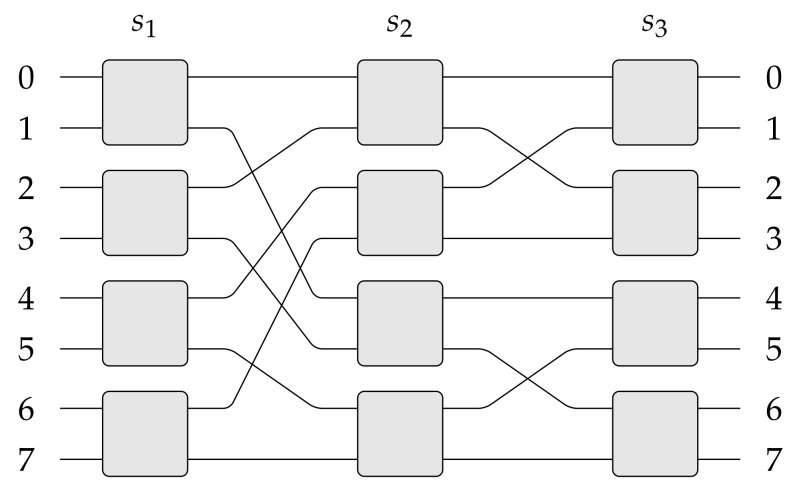
The *banyan* switching fabric of 8 inputs and 8 outputs (also called the $\log_{2}8$ switching fabric).

The *banyan* structure (see Figure 1), well known in the switching theory [5,6], as well as similar structures [7], are commonly used in optical networks. Thus, improvement of already known structures or the introduction of new switching fabrics is always welcomed, mainly when they help improve some parameters or decrease the cost of used switching nodes. The cost is very often given in the number of cross points [8,9,10,11,12,13,14].

**Figure 2:**
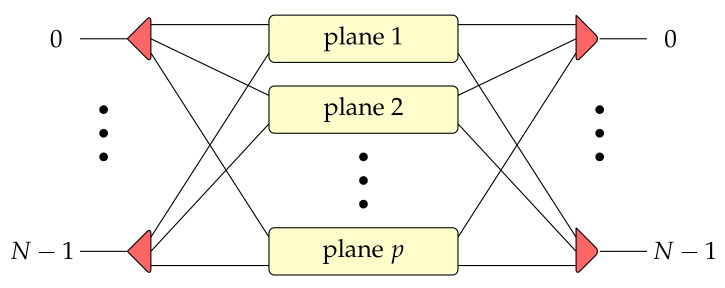
The idea of a multiplane switching fabric.

As the competitive structure to the *banyan*-type switching fabric, the $\log_{2}N-1$ switching fabric was introduced in [15], and later it was formally described in [16], where *N* denotes the number of inputs/outputs of switching fabric. It was shown that the $\log_{2}N-1$ switching fabric is a better solution for optical switching than the *baseline* switching network [17], where *baseline* constitutes a permutation pattern used in the *banyan* switching network. The multiplane version of the mentioned switching architecture is called the multi-$\log_{2}N-1$ switching fabric, and it was described in detail in [16] as well. A multiplane multi-$\log_{2}N-1$ switching network is achieved by vertically stacking *p* copies of the $\log_{2}N-1$ structure. The general idea of the multiplane (in general, *p*-plane) switching network is shown in Figure 2.

The exact number of planes *p* used to build the multi-$\log_{2}N-1$ switching fabric or the *baseline* switching fabric depends strongly on the type of nonblocking conditions, i.e., the strict-sense nonblocking (SSNB), the wide-sense nonblocking (WSNB), and the rearrangeable nonblocking (RRNB) conditions. The SSNB and the RRNB conditions for the space-division multi-$\log_{2}N-1$ switching fabric were described in detail and proved in [16]. The multi-$\log_{2}N-1$ switching network was later extended to the modified baseline architecture (MBA) switching fabric, whereas the SSNB and RRNB conditions for this switching network were delivered in [12] and [18], respectively. In a more general case, such a network is called the MBA(N,e,2) switching fabric. The *N* is the capacity of this switching network, *e* is the maximal number of inputs or outputs one switching element can have, and two means it is possible to extend this switching fabric to a structure of two-times greater capacity (number of inputs/outputs). For details about the MBA(N,e,2) switching fabric see [12].

Compared to to a *banyan*-type switching network, the [16] paper showed that the $\log_{2}N-1$ switching fabric is a desirable and promising solution. The solution’s attractive element is the cost of switching fabric, where the cost is expressed as the number of active and passive optical switching elements used to build such a switching network. For the *banyan*-type, the $\log_{2}N-1$, and the MBA(N,e,2) switching fabrics, not only the cost was discussed. The quality of optical signals which are sending out of these structures was investigated as well. This quality of the optical signal is very often expressed as the optical signal-to-crosstalk ratio (OSXR), and it is given in dB.

Recently, a new space switching structure was proposed by the author in [19]. This structure is called the MBA(N,e,g) switching fabric and it can be used in optical networks, for example, in the optical cross-connect (OXC), optical add-drop multiplexer (OADM), reconfigurable optical add-drop multiplexer (ROADM) circuit switching [20], in the space division multiplexing (SDM) switch [7], in the data center networks (DCNs) [21,22], or multiprocessor systems [23,24,25,26]. Using a new type of switching network structure in DCNs or multiprocessor systems allows building more energy-efficient and cheaper architectures. However, the topic of energy-efficiency switching fabrics is not considered in this study and it will be discussed in a future article. Nevertheless, such OSXR was not investigated before for the MBA(N,e,g) switching fabric introduced in [19]. Therefore, in this article, it is discussed OSXR for the MBA(N,e,g) switching fabric architecture, which can be extended not only to networks of 2-times greater capacity but to switching fabric of *g*-times greater capacity. The author presented previous work about the MBA(N,e,g) structure in [19] and put the main focus on the cost and optimization aspects only. In this study, the main focus is put on the OSXR in the MBA(N,e,g) switching fabric. It allows for the determination of the quality of the output optical signal in the MBA(N,e,g) structure and to compare it to the well known in switching theory *banyan*-type switching network. Therefore, this paper constitutes an extended version of the conference paper [19].

The remainder of this paper is organized as follows. In Section 2 the structure of the MBA(N,e,g) switching fabric is shortly described. In Section 3, the OSXR is described. In turn, in Section 4 achieved results are presented and compared with other switching fabrics for the same capacity and functionality. The last section constitutes conclusions.

## 2. Switching Fabric Architecture

**Figure 3:**
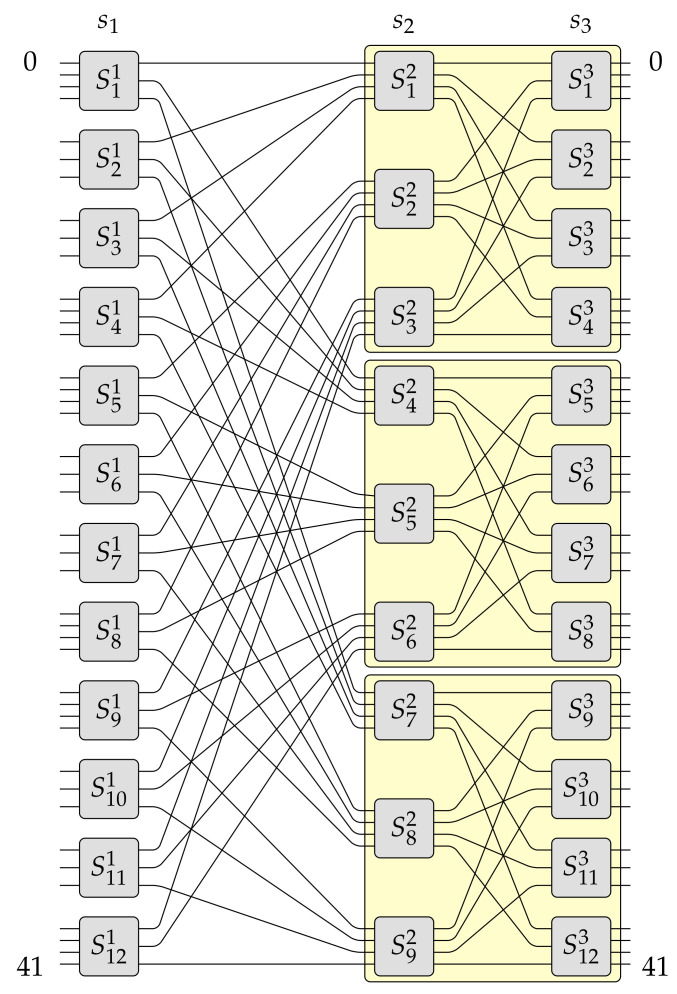
The MBA(42,4,3) switching fabric.

This section constitutes only a short description of the MBA(N,e,g) switching fabric architecture introduced in details in [19]. The *N* is the number of inputs and outputs of this switching fabric (also called capacity), *e* is the highest number of inputs (outputs) which one optical switching element (OSE) in the input (output) stage can have, and *g* denotes how many copies are used to build switching fabric of greater capacity. It is valid that 2⩽g⩽e. Inputs and outputs of the MBA(N,e,g) switching fabrics are numbered form 0 to N−1. An example of the MBA(N,e,g) switching fabric for N=42, e=4, and g=3 is shown in Figure 3. For more details about the the MBA(N,e,g) switching fabric, please refer to paper [19].

**Figure 4:**
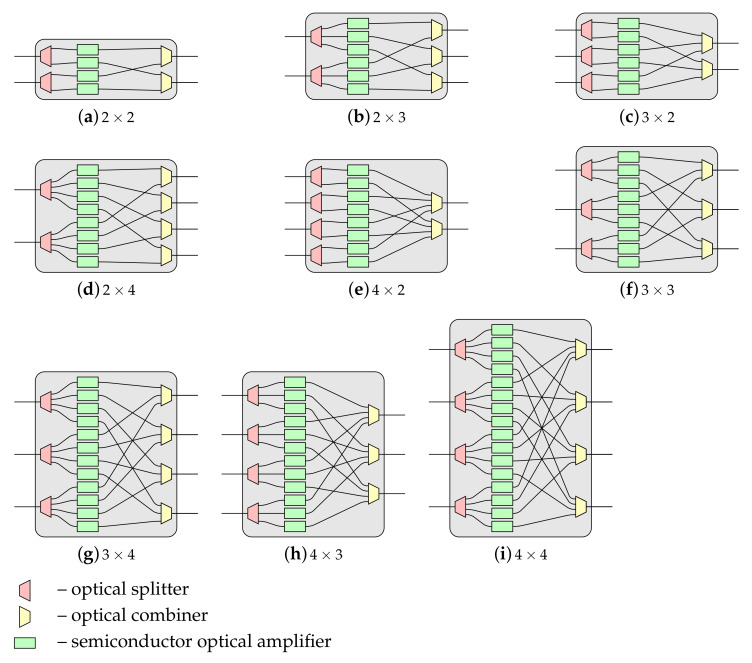
Optical switching elements (OSE) of different sizes.

The MBA(N,e,g) switching fabric architecture is a more general case of multi-$\log_{2}N-1$ and MBA(N,e,2) structures previously published by the author in [12,16], respectively. In contrast to the *baseline* switching network, the author’s recently proposed switching fabric is not only built of symmetrical OSEs but also and primarily of asymmetrical OSEs. Exemplary asymmetrical OSEs of sizes 2×2, 2×3, 3×2, 2×4, 4×2, 3×3, 3×4, 4×3, and 4×4 are shown in Figure 4. As can be seen, each OSE is built of smaller optical elements, i.e., passive optical elements like splitters and combiners and active optical elements like semiconductor optical amplifiers (SOAs). In Figure 4, optical splitters are marked in red, SOAs are marked in green, and in yellow are denoted optical combiners.

In general, the number of optical elements used to build an OSE of size x×y is *x* for optical splitters of size 1×y, *y* for optical combiners of size x×1, and x·y for SOAs.

The smallest possible capacity of an author’s recently proposed structure is called the base capacity, and it is denoted as:

(1) $$N_{0}=e^{2}-e+2.$$

**Figure 5:**
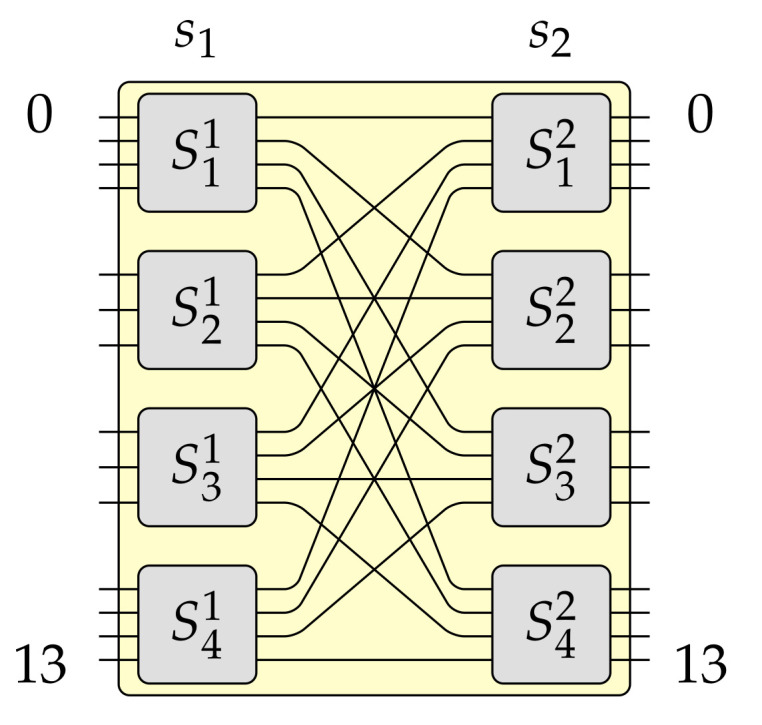
The MBA(14,4,g) switching fabric for g=2, g=3, and g=4.

For example, when e=3, e=4, or e=5 the base capacity is $N_{0}=8$, $N_{0}=14$, or $N_{0}=22$, respectively, and so on. The MBA$\left(N_{0},e,g\right)$ switching fabric, it means a switching fabric of the base capacity, has only two stages denoted as $s_{1}$ and $s_{2}$ (see Figure 5).

The number of stages in the MBA(N,e,g) switching fabric is denoted as:

(2) $$n=\left\lceil \log_{g}\left(\frac{N}{N_{0}}\right)+2\right\rceil$$

and these stages are called $s_{1}$, $s_{2}$, …, $s_{n-1}$, $s_{n}$. It should be noted that stages $s_{1}$ and $s_{n}$ are the outer stages, and they are always present independently of the capacity of the MBA(N,e,g) switching fabric. Other stages are called the inner stages, and their presence depends on the value of *n*. The capacity of a larger structure is:

(3) $$N=g^{i}N_{0},$$

where *i* is an integer value and i=0,1,2,…. The given capacity *N* of the MBA(N,e,g) switching fabric influences also the maximum number of connections established simultaneously. Due to differences in sizes of OSEs used to construct a particular architecture, such maximum number of simultaneously established connections is, of course, less than *N*. However, adding additional stages or additional planes (i.e., SSNB, WSNB, or RRNB conditions) can solve this issue, which is not the topic of this paper. Thus a switching fabric of a capacity $gN_{0}$ is denoted as MBA$\left(gN_{0},e,g\right)$. In the same way, it is possible to achieve another structure, i.e., the MBA$\left(gN_{0},e,g\right)$ switching fabric is a starting point to build the MBA$\left(g^{2}N_{0},e,g\right)$ structure, which is, in turn, a starting point to achieve the MBA$\left(g^{3}N_{0},e,g\right)$ switching fabric and so on and on. In general, it is possible to describe the way of constructing the MBA(N,e,g) switching fabric of *g* times greater capacity using an extension algorithm. Such an algorithm was introduced in [19], and here it is given as Algorithm 1.
**Algorithm 1** Constructing the MBA(N,e,g) switching fabric**Input**: The MBA$\left(\frac{N}{g},e,g\right)$ structure which will be extended
**Output**: The new MBA(N,e,g) structure of *g* times greater capacity
1Remove e−g OSEss of size (e−1)×a from the first stage of the MBA$\left(\frac{N}{g},e,g\right)$ switching fabric, where $$a=\left\{\begin{array}{cc} e & \mathrm{for}\;n=2\\ g & \mathrm{for}\;n⩾3 \end{array}\right..$$2Remove all interstage links between OSEs removed in step 1 and OSEs in the second stage and remove all not connected inputs (used earlier to connect to OSEs removed in step 1) from OSEs in the second stage.4If it is required, add a proper number of inputs to OSEs in the first stage that all OSEs in the first stage have right now *e* inputs.4Make g−1 copies of the switching fabric obtained in previous steps and put them below. This new structure has right now *N* outputs.5Add a new input stage which is the mirror image of the output stage—relevant OSEs are mirrored to each other. This newly added stage constitutes right now the first stage of the newly creating MBA(N,e,g) switching fabric.6Connect the outputs of the first stage OSEs’ to the proper inputs of the second stage switches’ using the *perfect unshuffle* pattern [5].


**Figure 6:**
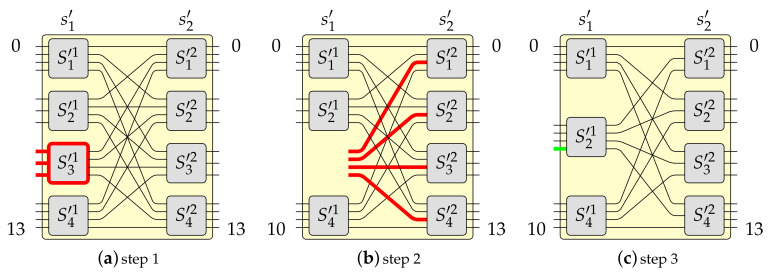
Algorithm 1—steps 1–3.

**Figure 7:**
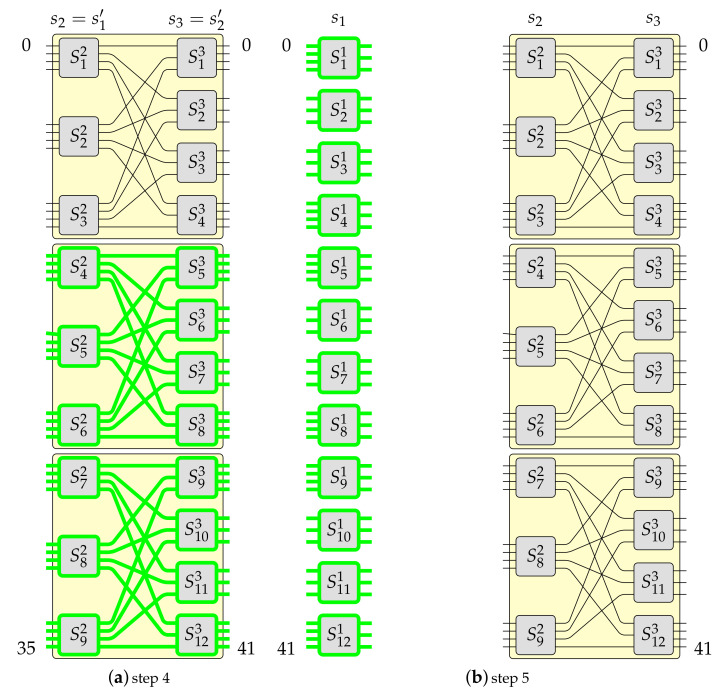
Algorithm 1—steps 4–5.

**Figure 8:**
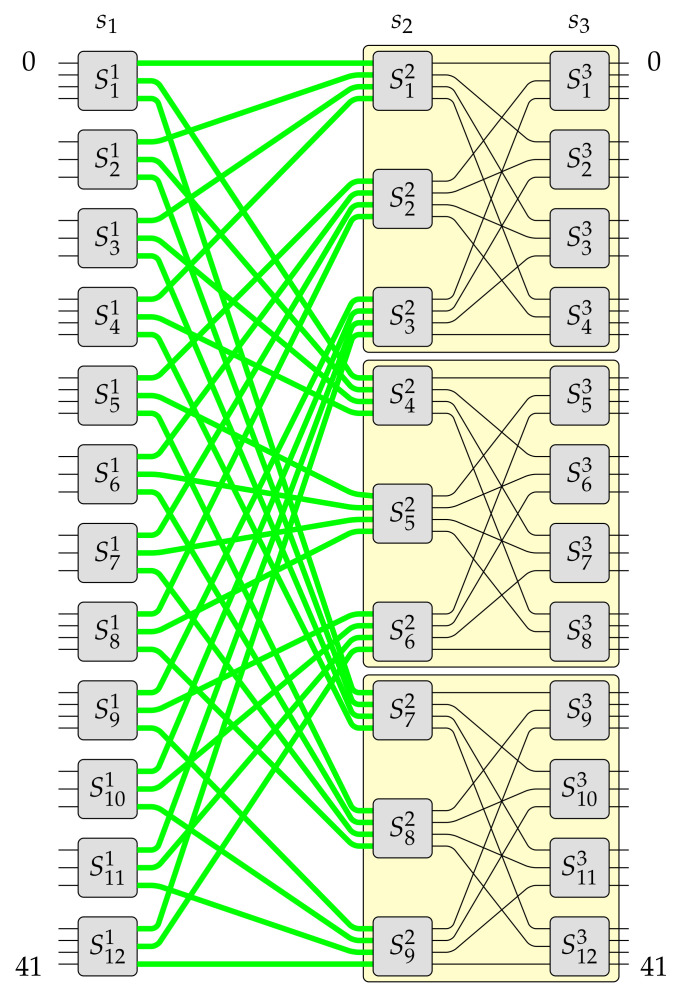
Algorithm 1—step 6.

Pursuant to Algorithm 1 it can be build a switching fabric of capacity $N=gN_{0}$, where 2⩽g⩽e. This example was earlier described in [19]. However, this time it is supported by figures presenting each step. Let us assume that in this example e=4 and g=3. The constructed MBA(42,4,3) switching fabric is shown in Figure 3. This switching fabric is obtained step-by-step, as it was shown in Figure 6, Figure 7 and Figure 8 (the red color denotes removed elements, the green color represents added elements), in the following way. Firstly, one OSE of size 3×4 (denoted in Figure 6a as $S_{3}^{\prime 1}$, where the upper index denotes stage’s number and bottom index denotes OSE’s number) is removed from the input stage in the MBA(14,4,3) structure (step 1 of Algorithm 1–see Figure 6a). In step 2, the proper interstage links and unused inputs from OSEs in the second stage are removed (see Figure 6b). Then an additional e−(e−1)=1 input to the relevant OSE in the first stage ($S_{2}^{\prime 1}$) is added (step 3 of Algorithm 1—see Figure 6c). The temporary structure has now twelve inputs and fourteen outputs. According to step 4 of Algorithm 1, such a structure is copied g−1=2 times, so the resulted switching fabric has thirty-six inputs and forty-two outputs (see Figure 7a). In the next step, a new stage from the switching fabric’s input side is added (see Figure 7b). The newly added input stage is a mirror image of a structure’s output stage achieved due to step 4. In the last step of Algorithm 1 (see Figure 8), the input stage is connected to other parts of the switching fabric of size 36×42 in accordance with the *perfect unshuffle* interconnection pattern [5]. As it can be seen, Figure 8 is exactly the same as Figure 3.

## 3. Crosstalk

In optical switching, each connection established in the switching fabric represents an optical signal which goes through stages. As it was already mentioned in Section 2 each stage is built from OSEs and each OSE is built from passive as well as active optical elements. Therefore, an optical signal goes through some number of optical splitters, optical combiners, and SOAs. This number depends strongly on the capacity of the switching network and its architecture.

**Figure 9:**
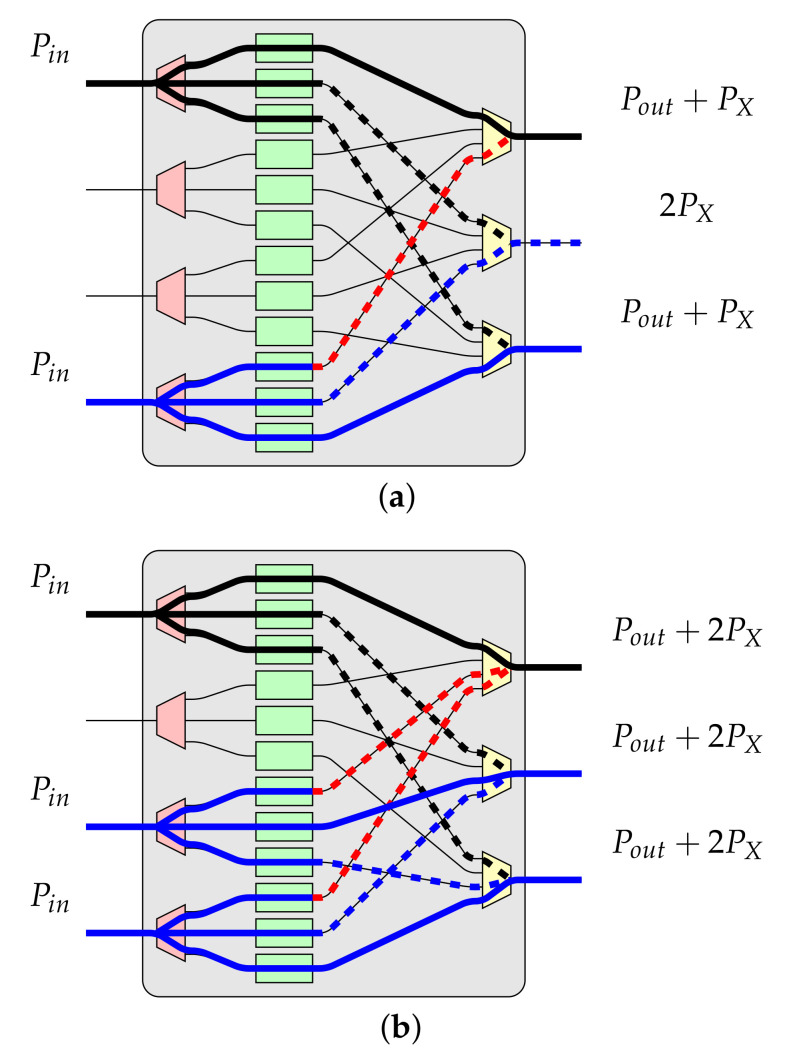
The optical crosstalk (dashed) in the OSE of size 4×3. (**a**) with the considered connection (solid black) and one intersecting connection (solid blue); (**b**) with the considered connection (solid black) and two intersecting connections (solid blue).

In this article, it was assumed that in one OSE, an optical signal goes through only one optical splitter, one SOA, and one optical combiner (see Figure 9). It was also assumed that an optical signal at the input to the OSE has an optical power $P_{in}$, and one SOA compensates all optical losses which appear inside this OSE. Therefore, the power of an optical signal at the OSE’s output is $P_{out}$, and it is, in the estimation, equal to the input signal’s optical power. Thus:

(4) $$P_{out}\approx P_{in}.$$

However, there is also some optical crosstalk that arises in each OSE through which some optical connection goes. Such optical crosstalk is, in fact, a small part of the optical power that influences other signal(s) already set up in the same OSE. This optical crosstalk is denoted as:

(5) $$P_{X}=mP_{in},$$

where usually m=0.01 which means that there is |X|= 20 dB loss between $P_{X}$ and $P_{in}$ [27,28].

An example of optical crosstalk is shown in Figure 9. It can be seen that optical crosstalk (denoted by the dashed bold line) from some input influences another connection (denoted by the solid bold line) and in result an optical power of the noise is added to the optical power of some connection, i.e., output signal’s power is $P_{out}=P_{in}+P_{X}$ or $P_{out}=P_{in}+2P_{X}$ (see Figure 9).

In Figure 9 an example of optical crosstalk in OSE of size 4×3 is shown. The solid black color line denotes the considered connection, and the solid blue color line represents other connection(s) established in the same OSE. The optical crosstalk originates from other connections, and interacting with the considered connection (the black connection) is denoted by the dashed bold red line. Another optical crosstalk is denoted by the dashed blue or black bold line, depending on its originates, and it is not interacting with the considered connection but with other connections. Such optical crosstalk is often called noise because it influences other “useful” connections, weakening them. Of course, in OSEs of different sizes, a similar situation can be observed.

As it can be seen in Figure 9, the maximal number of intersecting connections depends on the size of the OSE. In general, as it was assumed earlier in this article, the size of OSE is x×y. Therefore, if the number of inputs *x* in a particular OSE is equal or greater than the number of its outputs *y* (i.e., x⩾y), the maximal number of intersecting connections in such OSE is y−1. In turn, when the number of inputs *x* in a particular OSE is equal to or less than the number of its outputs *y* (i.e., x⩽y), the maximal number of intersecting connections in such OSE is x−1. For example, in OSE of size 4×3 there could be established a maximum of three connections. The first connection is the considered connection (denoted by the solid bold black line in Figure 9b), and two are the intersecting connections (marked by the solid bold blue line in Figure 9b). More connections are not possible due to the lack of free outputs, even if there is one free input. A very similar situation is when x⩽y.

In the *banyan*-type optical switching network each OSE is symmetrical and it has size d×d. Therefore, in the worst-case, there can be d−1 optical crosstalk per stage. There are $\log_{d}N$ stages, thus the OSXR is:

(6) $$OSXR_{\log_{d}N}=\left|X\right|-10\log_{10}\left(\left(d-1\right)\log_{d}N\right)\;\left[\mathrm{dB}\right].$$

In the MBA(N,e,g) switching fabric OSEs have a different sizes depending on the stage where they are localized (see Figure 3). Therefore, in one OSE in the worst-case, there could be between only one to g−1 or e−1 optical crosstalks. There is only one crosstalk when establishing the considered connection and only one other connection—so-called the intersecting connection. Such situation is valid, for example, for OSEs of size 2×2, 2×3, and 3×2. There are more crosstalks when there are more intersecting connections (see Figure 9b). The exact maximal number of crostalks in a particular OSE depends on the size of a particular OSE, i.e., it could be e−1 or g−1. Moreover, the number of optical crosstalk depends on the number of stages *n*, which results from the capacity *N* of the MBA(N,e,g) switching fabric. In general, the MBA(N,e,g) structure has *n* stages. Thus, there could be distinguished three cases. The first case is valid for n=2 stages:

(7) $$OSXR_{MBA(N,e,g)}^{n=2}=\left|X\right|-10\log_{10}\left(2e-2\right)\;\left[\mathrm{dB}\right],$$

the second case is for n=3 stages:

(8) $$OSXR_{MBA(N,e,g)}^{n=3}=\left|X\right|-10\log_{10}\left(2g+e-3\right)\;\left[\mathrm{dB}\right],$$

and the third case is for a greater number of stages (n⩾4):

(9) $$OSXR_{MBA(N,e,g)}^{n⩾4}=\left|X\right|-10\log_{10}\left(n(g-1)\right)\;\left[\mathrm{dB}\right].$$

Let us take a closer look at some examples of how to calculate the crosstalk for the MBA(N,e,g) switching fabric when the number of stages is n=2. The parameters of this network are: N=14, e=4, and g=2 (see Figure 5). This means, according to Equations (1) and (2), there are $n=\left\lceil \log_{2}\left(\frac{14}{4^{2}-4+2}\right)+2\right\rceil =\left\lceil \log_{2}\left(\frac{14}{14}\right)+2\right\rceil =\left\lceil \log_{2}\left(1\right)+2\right\rceil =2$ stages. In the first stage (stage $s_{1}$), in the worst-case, there will be e−1=4−1=3 intersecting connections, because there are OSEs of sizes 4×4 and 3×4 (i.e., e×e and e−1×e, respectively) and the maximal number of intersecting connections from values e−1=3 (in OSE of size 4×4 there could be established three connections simultaneously) and e−2=2 (in OSE of size 3×4 there could be established two connections simultaneously) is e−1=3. In the last stage (stage $s_{2}$), in the worst-case, there are OSEs of sizes 4×4 and 4×3 (i.e., e×e and e×e−1, respectively). Thus, the maximal number of intersecting connections is e−1. Summarizing all the above together, the $OSXR_{\mathrm{MBA}(14,4,2)}$ can be calculated. So,

(10) $$\begin{array}{cc} OSXR_{\mathrm{MBA}(14,4,2)}^{n=2}\; & =\left|X\right|-10\log_{10}\left((e-1)+(e-1)\right)\\ \; & =\left|X\right|-10\log_{10}\left(2e-2\right)\\ \; & =\left|X\right|-10\log_{10}\left(6\right)=12.2185\;\left[\mathrm{dB}\right], \end{array}$$

which gives exactly the same formula that is given by Equation (7).

Let us take a closer look at another example of how to calculate the crosstalk for the MBA(N,e,g) switching fabric when the number of stages is n=3. The parameters of this network are: N=42, e=4, and g=3 (see Figure 3). This means, according to Equations (1) and (2), there are $n=\left\lceil \log_{3}\left(\frac{42}{4^{2}-4+2}\right)+2\right\rceil =\left\lceil \log_{3}\left(\frac{42}{14}\right)+2\right\rceil =\left\lceil \log_{3}\left(3\right)+2\right\rceil =3$ stages. In stage $s_{1}$, in the worst-case, there will be g−1=3−1=2 intersecting connections in any OSE, because there are OSEs of sizes 4×3 and 3×3 (i.e., e×g and e−1×g, respectively) and the maximal number of intersecting connections is g−1=2 (there are always *g* outputs in any OSE in stage $s_{1}$). In the second stage (stage $s_{2}$), in the worst-case, there are only OSEs of size 4×4 (i.e., e×e). Therefore, the maximal number of intersecting connections in any of these OSEs is always e−1=3. In the last stage (stage $s_{3}$), in the worst-case, there are OSEs of sizes 3×4 and 3×3 (i.e., g×e and g×e−1, respectively). Thus, the maximal number of intersecting connections is g−1=2. Taking all the above together, the $OSXR_{\mathrm{MBA}(42,4,3)}$ can be calculated. So,

(11) $$\begin{array}{cc} OSXR_{\mathrm{MBA}(42,4,3)}^{n=3}\; & =\left|X\right|-10\log_{10}\left((g-1)+(e-1)+(g-1)\right)\\ \; & =\left|X\right|-10\log_{10}\left(2g+e-3\right)\\ \; & =\left|X\right|-10\log_{10}\left(7\right)=11.5490\;\left[\mathrm{dB}\right], \end{array}$$

which gives exactly the same formula that is given by Equation (8).

Let us take a look closer at the last example. In this case, it is shown how to calculate the crosstalk for the MBA(N,e,g) switching fabric, which has n⩾4 stages. The parameters of this network are: N=126, e=4, and g=3. This means, according to Equations (1) and (2), there are $n=\left\lceil \log_{3}\left(\frac{126}{4^{2}-4+2}\right)+2\right\rceil =\left\lceil \log_{3}\left(\frac{126}{14}\right)+2\right\rceil =\left\lceil \log_{3}\left(9\right)+2\right\rceil =4$ stages. In stage $s_{1}$, in the worst-case, there will be g−1=3−1=2 intersecting connections in any OSE, because there are OSEs of sizes 4×3 and 3×3 (i.e., e×g and e−1×g, respectively) and the maximal number of intersecting connections is g−1=2 (there are always *g* outputs in any OSE in stage $s_{1}$). In the second stage (stage $s_{2}$), in the worst-case, there are only OSEs of size 4×3 (i.e., e×g). Therefore, the maximal number of intersecting connections in any of these OSEs is g−1=2. In the third stage (stage $s_{3}$), in the worst-case, there are only OSEs of size 3×4 (i.e., g×e). Therefore, the maximal number of intersecting connections in any of these OSEs is g−1=2. In turn, in the last stage (stage $s_{4}$), in the worst-case, there are OSEs of sizes 3×4 and 3×3 (i.e., g×e and g×e−1). Thus, the maximal number of intersecting connections is g−1=2. Taking all the above together, the $OSXR_{\mathrm{MBA}(126,4,3)}$ can be calculated. So,

(12) $$\begin{array}{cc} OSXR_{\mathrm{MBA}(126,4,3)}^{n=4}\; & =\left|X\right|-10\log_{10}\left((g-1)+(g-1)+(g-1)+(g-1)\right)\\ \; & =\left|X\right|-10\log_{10}\left(n(g-1)\right)\\ \; & =\left|X\right|-10\log_{10}\left(8)\right)=10.9691\;\left[\mathrm{dB}\right], \end{array}$$

which gives exactly the same formula that is given by Equation (9).

Similarly, the OSXR for the MBA(N,e,g) of different parameters could be calculated.

## 4. Results

**Figure 10:**
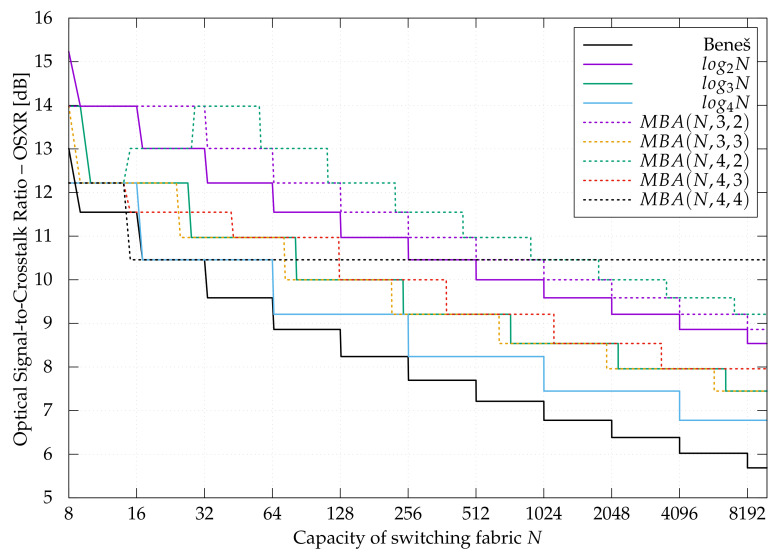
Optical signal-to-crosstalk Ratio (OSXR) for different switching fabrics.

The OSXR for different structures is compared in Figure 10. It can be seen that the MBA(N,e,g) switching fabric is almost always better (sometimes equal) OSXR than reference Beneš [27,28] or *banyan*-type switching network. The only exception is for N=8 when $\log_{2}8$ has the highest OSXR. Using a recently proposed MBA(N,e,g) switching fabric structure allows for the improvement of the output optical signal’s quality. This is very important in optical networking because any kind of regeneration between switching nodes (in optical links/fibers) is costly.

Let us take a look closer at some examples for switching networks of capacity N=128. The OSXR of:

- Beneš is $OSXR_{Benes}=$ 8.8606 dB,
- $\log_{2}128$ is $OSXR_{\log_{2}128}=$ 11.5490 dB,
- $\log_{3}128$ is $OSXR_{\log_{3}128}=$ 10.0000 dB,
- $\log_{4}128$ is $OSXR_{\log_{4}128}=$ 9.2082 dB,
- MBA(128,3,2) is $OSXR_{\mathrm{MBA}(128,3,2)}=$ 12.2185 dB,
- MBA(128,3,3) is $OSXR_{\mathrm{MBA}(128,3,3)}=$ 10.000 dB,
- MBA(128,4,2) is $OSXR_{\mathrm{MBA}(128,4,2)}=$ 12.2185 dB,
- MBA(128,4,3) is $OSXR_{\mathrm{MBA}(128,4,3)}=$ 10.0000 dB,
- MBA(128,4,4) is $OSXR_{\mathrm{MBA}(128,4,4)}=$ 10.4576 dB.

It can be clearly seen that for capacity N=128, the best OSXR in dB has the MBA(128,3,2) and the MBA(128,4,2) switching fabrics (the OSXR is exact 12.2185 dB). Both structures are switching fabric recently proposed in [19]. The OSXR for other compared switching networks for this same capacity is lower (see Figure 10).

It should be noted that the MBA(N,e,g) switching fabric was not designed to be the best one in terms of OSXR but to be cheaper than the traditional *banyan*-type structure (the competitor structure). It could be said that the MBA(N,e,g) network is an improved version of the *baseline* switching fabric. Designing switching fabric, which is the best one in many aspects, is very difficult, if even impossible because it depends on many factors and places where such structure will be used. Thus, for different purposes, different structures are used. It is also the reason the MBA(N,e,g) structure was not compared, for example, to the Dilated Beneš architecture, which was designed specifically to improve the quality of the optical signal at the switching node output. From the point of view, where the cost (expressed in the number of optical elements) is the most important metric, the Dilated Beneš network is not the switching fabric of the first choice because it is more expensive (it is built from a greater number of optical elements). Here we can talk about the multicriteria optimization problem, which is not the topic of this paper.

## 5. Conclusions

The MBA(N,e,g) optical switching fabric, introduced in the author’s conference paper, is the most general case of the $\log_{2}N-1$ and the MBA(N,e,2) networks. In contrast to the *banyan*-type switching network, the MBA(N,e,g) structure fabric requires a lower number of passive as well as active optical elements, which was given in [19]. Moreover, the MBA(N,e,g) switching network is built from asymmetrical as well as symmetrical optical switching elements in contrast to the typical *banyan*-type switching network, which is built only from symmetrical optical switching elements. In comparison to the typical *banyan*-type switching network, the MBA(N,e,g) switching fabric of the same capacity and functionality is built from a smaller number of stages. It caused the optical signal to pass through a few less optical elements, i.e., it may influence the signal quality expressed by attenuation or by crosstalk ratio. This question was open, and this paper finally answered it. During the investigation in this study, this influence was expressed by the OSXR. It was shown that the recently proposed MBA(N,e,g) architecture has almost always a stronger output’s optical signal for any capacity *N* than a relevant *banyan*-type network of the same capacity and functionality. Of course, the stronger the optical signal at the output of the switching network means better quality of transmitted data. When the optical signal has greater optical power, it can be transmitted for longer distances without regeneration. Such possibility is very desirable in optical communication since any regeneration is costly. Therefore, all mentioned aspects make the MBA(N,e,g) switching fabric a desirable solution for optical networking.

It should be noted that this paper delivered only the OSXR for the MBA(N,e,g) switching fabric and compared it with the *banyan*-type switching network. This is because the MBA(N,e,g) switching fabric was designed as a straight update of the *banyan*-type network. The achieved results clearly show that the new switching fabric is indeed the updated version of the *banyan*-type architecture in terms of the cost and the quality of the outputs optical signals. Other switching network architectures, designed especially for better OSXR, were not compared in this paper. They have greater cost (expressed in the number of optical elements) than the *banyan*-type network or the MBA(N,e,g) switching fabric. However, such comparison is also possible; this became the multicriteria optimization problem, which is not the topic of this study.

## Data Availability

Not applicable.

## Abbreviations

The following abbreviations are used in this manuscript:

DCN	data center network
MBA	modified baseline architecture
OADM	optical add-drop multiplexer
OSE	optical switching element
OSXR	optical signal-to-crosstalk ratio
RRNB	rearrangeable nonblocking
SDM	space division multiplexing
SOA	semiconductor optical amplifier
SSNB	strict-sense nonblocking
VNI	visual networking index
